# Assessment of the impact of availability and readiness of malaria services on uptake of intermittent preventive treatment in pregnancy (IPTp) provided during ANC visits in Tanzania

**DOI:** 10.1186/s12936-019-2862-3

**Published:** 2019-07-09

**Authors:** Shraddha Bajaria, Charles Festo, Sigilbert Mrema, Josephine Shabani, Ellen Hertzmark, Ramadhani Abdul

**Affiliations:** 1 Ifakara Health Institute, Box 78373, Dar es Salaam, Tanzania; 2 Department of Global Health and Population, Harvard TH Chan School of Public Health, Boston, MA USA

**Keywords:** Tanzania, Malaria, IPTp, Service readiness, Service availability, Health services

## Abstract

**Background:**

Intermittent preventive treatment during pregnancy (IPTp) is a highly-recommended intervention to prevent maternal and neonatal complications associated with malaria infection. Despite fairly high antenatal care (ANC) coverage in Tanzania, low IPTp uptake rates represent a gap in efforts to decrease complications attributed to malaria in pregnancy. The objective of this study was to examine if availability, readiness and managing authority are associated with uptake of IPTp during ANC.

**Methods:**

Data for this analysis come from a cross-sectional survey, the Tanzania Service Provision Assessment conducted between 2014 and 2015. Principal component analysis was used to create scores for availability of malaria services and readiness for the provision of services. Generalized estimating equation models with logit link and the binomial distribution assessed factors that impact the uptake of IPTp by pregnant women attending ANC.

**Results:**

Higher fraction of women in their third trimester than second (68% versus 49%, OR = 2.6; 95% CI (2.1–3.3)), had received at least one dose of IPTp. There was a wide variation in the availability and readiness of malaria services provision and diagnostic tools by managing authorities. Public facilities were more likely than private to offer malaria rapid diagnostic test, and more providers at public facilities than private diagnosed and/or treated malaria. Women who attended facilities where direct observation therapy was practiced were more likely to have received at least one dose of IPTp (64% versus 46% who received none; p < 0.001). Women who attended ANC at a facility with a high readiness score were more likely to take IPTp than those attending facilities with low readiness scores (OR = 2.1; 95% CI (1.4–3.3)). Reported stock out on the day of interview was negatively associated with IPTp uptake (OR 0.09; 95% CI 0.07–0.1).

**Conclusion:**

Readiness of health facilities to provide malaria related services, the number of ANC visits and gestational age were associated with uptake of IPTp among women attending ANC. There are disparities in malaria service availability and readiness across geographical location and managing authorities. These findings could be used to assist the malaria programme and policymakers to appropriately decide when planning for malaria service deliveries and interventions.

**Electronic supplementary material:**

The online version of this article (10.1186/s12936-019-2862-3) contains supplementary material, which is available to authorized users.

## Background

Pregnancy-associated malaria remains a major concern in many malaria-endemic areas, although it is preventable and treatable [1]. The most commonly reported effects of pregnancy-associated malaria include stillbirth, fetal anaemia, low birth weight and preterm delivery [2, 3]. Up to 20% of stillbirths in sub-Saharan Africa result from malaria in pregnancy [4]. Low birth weight is a major risk factor for infant mortality [5]. In 2004, it caused about 100,000 infant deaths in Africa [6]. In Tanzania, where maternal mortality is extremely high (556 deaths per 100,000 women in 2018) [7], an estimated 1.7 million pregnant women are at risk of malaria infection every year [8]. Many collaborations between malaria and reproductive health programmes are formed to deliver the World Health Organization’s (WHO) recommended guidelines of providing at least three doses of sulfadoxine–pyrimethamine (SP) as intermittent preventive treatment during pregnancy (IPTp), promoting the use of insecticide-treated nets (ITNs) and adequate diagnosis, management and treatment of malaria in pregnant women, to address the challenges through standard antenatal care (ANC) [1].

Antenatal care for pregnant women by skilled health providers is important for maintaining a healthy pregnancy, improving pregnancy outcomes by identifying any complications, promoting healthy behaviors and providing clients with an opportunity to actively engage with health providers [9]. ANC coverage in sub-Saharan African countries has improved over the years, although the rates vary across countries [10, 11]. According to the 2015–2016 Tanzania Demographic and Health Survey (DHS), almost all pregnant women in Tanzania (98%) attended ANC at least once, but only half (51%) had four or more ANC visits as recommended by WHO. WHO recommends provision of IPTp at each scheduled antenatal visit in the second and third trimesters, with doses given at least 1 month apart, ideally as directly-observed treatment (DOT) [12]. Despite fairly high ANC coverage, uptake of IPTp by pregnant women remains low in Tanzania [13]. In 2012, Tanzania’s National Malaria Control Programme (NMCP) set a target of increasing the percentage of women reporting a live birth in the previous 2 years who received two doses or more of IPTp from 32% in 2012 to 80% in 2016 and continuing to 2020 [14]. However, the 2015–2016 DHS reported that the percentage of pregnant women who received at least two doses of IPTp in 2016 was 35% [15], though the Malaria Indicator Survey (MIS) 2017 showed that the percentage was 56% [16]. The wide gap between the national target and the achieved rates, regardless of multiple scale-up efforts, represents unexplored opportunities. In contrast to Tanzania mainland, Zanzibar is at a pre-elimination stage for malaria, after scaling up WHO’s recommended guidelines in 2006 [17]. Due to low prevalence of malaria in Zanzibar, IPTp is no longer given during ANC [18].

While numerous studies have examined and continue to reveal women’s individual factors; such as age, education level, receiving health educational information and HIV counseling during ANC, previous pregnancy complications [19], number of ANC visits [20], and history of pregnancy to be positively associated with the attendance of ANC and uptake of IPTp, fewer studies have examined factors at the facility and provider levels [21, 22]. Individual and system factors that have been negatively associated with ANC attendance are never being married, long distance to ANC facility, young age and limited pregnancy disclosure [1, 23]. Managing authority, defined as public or private, has also been independently associated with the uptake of IPTp [24]. Private facilities were less likely to administer IPTp than facilities managed by public authorities [25]. Geographical zones were also a factor in ANC attendance and uptake of IPTp by women [9]; residing in regions of Eastern and Coastal zone was significantly associated with higher uptake of IPTp compared to residence in any other zone [26]. Prevalence of malaria is the highest for regions in the Lake, West and Southern zones [18], the uptake of IPTp by women aged 15–49 years in these zones was low to moderate for women in Lake and West zones and relatively higher in Southern zone [16]. Higher household economic status has a positive influence on uptake of IPTp; women from non-poor households are more likely to attend ANC clinics [27]. Sociocultural practices such as religious beliefs or family support also influence ANC attendance, therefore IPTp uptake [27]. A qualitative study in Uganda identified inconsistent guidelines, lack of training and supervision, stock-out and weak supply chain as some of the supply-side barriers to uptake of IPTp by pregnant women [28]. The latest Service Availability and Readiness Assessment (SARA) report of 2017 indicated high availability of selected components of malaria services with differences across facility type, managing authority and facility location, IPTp was more often available in public facilities compared to private. Readiness for provision of some of these malaria services was lower; specifically, the proportion of staff trained in provision of IPTp, was much lower at private facilities than public [29].

Using TSPA data, this study aims to assess facility-based factors of provision of malaria services to pregnant women during ANC. Specifically, this study describes availability of malaria services and health facility and provider readiness to deliver these services by managing authority (public versus private), and analyses if availability, readiness and managing authority are associated with uptake of IPTp dose during ANC visits. The results will add to the limited existing literature on supply-side barriers and assist officials in identifying components of ANC that possibly hinder achievement of national target.

## Methods

The TSPA surveys use standardized data collection instruments, which are detailed in the 2014–2015 TSPA report [30]. TSPA surveys include four main questionnaires: facility inventory, client observation protocols, exit interviews and health provider interviews. For the current analysis, the facility inventory and the health provider questionnaires data were used to identify the services available and assess the general and specific service readiness at each facility. Interviewer observation protocols and client exit interviews for ANC were used to assess the clients’ perception of the services received. Selected supply-side service availability and facility readiness components (see “Details of measurement” below) were analysed to explore their effects on uptake of IPTp by pregnant women.

### Sampling

The TSPA survey used a master facility list representative of the country’s formal-sector facilities. TSPA had a list of 7102 health facilities, 6838 on the mainland and 264 in Zanzibar. A multi-stage sampling technique was used to first select the facilities randomly and thereafter select the health providers and clients for observation and exit interviews: 1200 health facilities (1090 from Mainland and 110 from Zanzibar) were selected. An average of eight health providers at each facility was interviewed; for facilities with fewer than eight providers available, all providers present were interviewed. ANC clients were identified systematically with a maximum of five clients per provider and 15 per facility were observed. Further details are explained in the 2014–2015 TSPA report [30].

### Details of measurement

The SARA manual [31] was used to identify variables for availability and readiness. Service availability is defined as the physical presence and sufficient supply of services at the facility and competent workforce for provision of services. Service readiness is defined as the capacity and ability of health facilities and providers to deliver the services [31]. The following variables from three data files were considered to assess the main outcome of interest; number of pregnant women who reported receipt IPTp doses.

#### Facility data

Facility type (national referral, regional, district, other hospital, health centre, clinic and dispensary), managing authority (public or private), region, services available, whether the facility offered any malaria or ANC services (including the diagnosis and treatment of malaria, the number of malaria and ANC providers available at the facility), and those who had received training, technical support and work supervision, availability of IPTp and national ANC guidelines, and whether SP was in stock at the facility.

#### Health provider data

Permission to interview, technical qualification of the provider, whether providers diagnosed and/or treated malaria, whether providers provided ANC care, whether providers conducted any malaria lab services, whether providers had received in-service training for diagnosing malaria and for providing IPTp to pregnant women.

#### Interviewer observations and exit interviews for ANC client data

Whether malaria rapid diagnostic test (RDT) is routinely provided, whether provider discussed the importance of at least four ANC visits, whether DOT for uptake of IPTp was available, whether explanations were given to the client regarding how to take an anti-malarial and the possible side effects of an anti-malarial, the importance of further doses of IPTp and the use of ITNs, the gestational age in weeks as indicated on the ANC card, and client characteristics such as age and education level.

### Data management

Since there is very low prevalence of malaria in Zanzibar and IPTp is no longer implemented, only facilities from mainland Tanzania were included in this analysis. Out of 1090 facilities, 12 (1.1%) did not respond, and other 129 (12%) did not offer any ANC services, leaving 949 (88%) for this analysis. From the sample of health providers who were present on the day of the visit, only the 6418 (98%) who consented to be interviewed and reported to provide any client services were included. Client–provider consultations were observed by the interviewers to assess the quality of services provided. In particular, interviewers observed whether protocols and national guidelines were followed and assessed whether providers were prepared to provide the required services. Personal characteristics of the clients were reported in the exit interview questionnaires. Only 3463 (99.6%) pregnant women who had gestational age in weeks indicated on their ANC cards were included in these analyses. Doses of IPTp received were indicated on women’s ANC cards, of which only those doses received IPTp at the facility where they were interviewed were included (95.5%).

Managing authority was defined as public versus private facilities, with parastatal categorized as public and mission/faith-based categorized as private facilities. The 25 mainland Tanzania regions were categorized into six zones as per ministry of health classifications; Central, Coastal, Lake, Northern, Southern Highlands and Western [32]. Gestational age was used to categorize women into trimesters, with women in their 1st to 13th weeks in the first trimester, 14th to 26th weeks in the second trimester and 27th to 44th weeks in the third trimester. The final analyses only included women in their second or third trimester, as Tanzania guidelines do not suggest IPTp uptake before the second trimester. The number of IPTp doses recorded on the ANC card was used to categorize women as not having received IPTp dose or having received at least one IPTp dose. Analysis was performed for both; including and excluding the IPTp dose received on the day of interview. Availability of SP at the facility was categorized into three; never available, available (observed non-expired SP and reported availability) and not available today (not available or invalid).

An availability score was created using the following variables: whether the facility offers any malaria tests, whether the facility offers diagnosis or treatment of malaria, whether providers diagnose and/or treat malaria, whether providers provide any ANC/PNC care, whether ANC providers conduct any malaria lab services, distance of the facility from the clients’ home, and whether the provider offered an ITN to client.

A readiness score was created using the following variables: observation of training manual/poster/job aid for RDT, whether the national guidelines for treatment of malaria observed, whether the provider had received training in diagnosing malaria in adults, providing IPT for pregnant women, and how to perform malaria microscopy, whether the provider had received any in-service training for ANC/PNC or any lab in-service training or malaria microscopy.

### Data analysis

Stata 15 software was used to analyse data for this study. Frequencies and proportions of selected availability and readiness indicators were described by managing authority. The main outcome of interest was whether an IPTp dose was received by the women for current pregnancy at the index facility, as indicated on their ANC cards. Generalized estimating equation (GEE) models with logit link and the binomial distribution were used to study the association between selected availability and readiness variables and doses of IPTp. Principal component analysis (PCA) was used to create scores for the availability of malaria services and readiness for the provision of malaria services, for which three tertiles were created (high, medium and low). An alpha value of p = 0.05 was used to determine the statistical significance for all analyses. Factors significant at p ≤ 0.05 in univariable model, confounders that were significantly associated in literature, along with variable of interest were added to form multivariable models. The odds ratio (OR) was used as a measure of association between exposure and outcome variables and is reported with its 95% confidence interval (CI). To take into account disproportionate sampling of facilities by regions a multiplier (weight) was used in all analyses in order to restore representativeness for over/under sampling. Health facility level (national referral, regional, district, another hospital, health centre, clinic and dispensary) was controlled for, in the multivariable analysis, to give a better estimate of the true difference between IPTp uptake at public and private facilities.

## Results

A total of 1078 health facilities from mainland Tanzania responded to the SPA 2014–2015, of which 949 (88.0%), 690 (95.5%) of all the public and 259 (72.9%) of all the private provided ANC services. A total of 6418 health providers [4011 (99.2%) of all public and 2407 (96.7%) of all private facilities] were interviewed. Nurse professionals made up the highest proportion of health providers in both public (63.3%) and private (57.1%) facilities. Consultations of 3478 ANC clients were observed, of which 2501 (71.9%) were at public facilities and 977 (28.1%) were at private facilities.

### Availability of malaria services at public and private facilities

Additional file 1 presents the weighted frequencies and proportions for variables of availability by managing authority. Availability of malaria tests and malaria RDT was higher in public facilities (86.8%) and (86.2%) than in private facilities (78.1%) and (71.0%) respectively. Only 1.4% of all facilities reported never having SP available, 40.7% reported not having SP on the day of interview while 57.9% reported having SP (58% of public facilities and 56% of private facilities). A higher proportion of providers at public facilities (77.6%) diagnosed or treated malaria than at private facilities (60.9%). However, a significantly higher proportion of providers at private facilities provided malaria microscopy (76.5%) compared to those at public facilities (33.7%). Malaria RDT was provided by 88.4% and 84.4% of health providers at public and private facilities respectively. A significantly higher proportion of providers at public facilities provided ANC/PNC care (75.8%) than at private facilities (40.5%). The fraction of providers who were medical doctors was almost twice as high in private facilities as in public ones (7.7% and 3.9%, respectively).

### Readiness of public and private facilities to provide malaria services

**Fig. 1:**
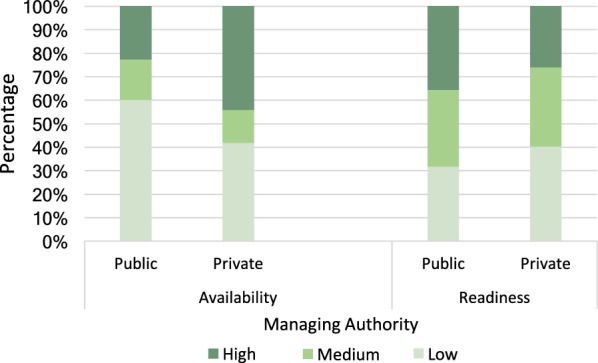
Malaria services availability and readiness by facility managing authority. A higher proportion (60%) of public facilities scored low for availability compared to private (42%, p < 0.001) while a higher proportion (40%) of private facilities scored low for readiness, compared to 32% for public (p = 0.14)

**Fig. 2:**
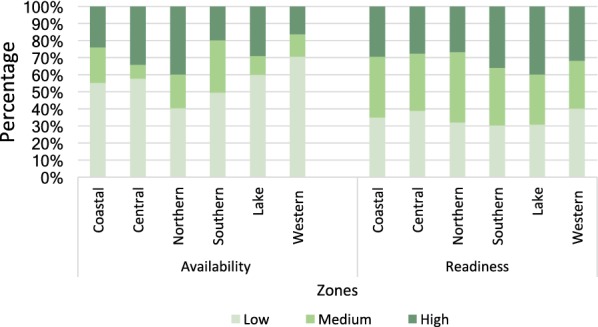
Malaria services availability and readiness by geographical zones. Malaria services vary in both readiness and availability across zones. The Western zone had the lowest availability score (30%, p < 0.001). There was little variation in facility readiness across zones (p = 0.67), with facilities in Southern and Lake zones scoring the highest (70%)

Additional file 1 also presents the weighted frequencies and proportions for variables of readiness by managing authority. A higher proportion of private facilities (43.2%) had a training manual or a job aid for using malaria RDT available, while the national guideline for the diagnosis and treatment of malaria was observed at a higher proportion of public facilities (62.8%). Forty-three percent (43.8%) of the providers at public facilities and 28.1% of the providers at private facilities had received in-service training about malaria, while a significantly higher proportion (18.9%, p < 0.001) of malaria service providers at private facilities received training on how to perform malaria microscopy. A higher proportion of providers received in-service training (30.8%) about the provision of IPTp (78.7%) during ANC at public facilities compared to those at private facilities. Slightly higher proportions of providers at public facilities gave SP during the consultation, explained the purpose of preventing malaria, explained how to take anti-malarials, and explained the importance of further IPTp doses and use of ITNs. However, direct observation of uptake of IPTp was higher at private facilities (70%) (Figs. 1, 2).

### Characteristics of women attending ANC services by IPTp status

Weighted frequencies and percentage for women’s ages and education level, by the doses of IPTp that they received are presented in Table 1. Receipt of IPTp did not vary by women’s age. Women who had not received any education were less likely to have received at least one dose of IPTp (52.6%) than those who had primary or secondary and above education (59.1% and 56.9%, p = 0.12). Slightly higher proportion of women in the urban area received IPTp dose than rural, the difference was not statistically significant. The proportion of women who had ever received at least one dose of IPTp was slightly higher in those attending ANC at private (59.8%) than public (56.7%) facilities, although this was not statistically significant. After excluding the IPTp dose received on the day of interview, the proportion of women who had at least one dose was 34.1% at public and 39.0% at private facilities (p = 0.19).

**Table 1:** Characteristics of women attending ANC services by IPTp status

	None		At least 1 dose of IPTp		Overall	p-value
	n	(%)	n	(%)		
Facility location						0.13
Urban	377	(38.2)	611	(61.8)	989	
Rural	1110	(44.5)	1385	(55.5)	2495	
Managing authority						0.55
Public	1214	(43.3)	1590	(56.7)	2803	
Private	274	(40.2)	407	(59.8)	681	
Age						0.08
15–24	746	(43.8)	959	(56.2)	1705	
25–35	600	(40.9)	865	(59.1)	1465	
35 above	111	(41.3)	158	(58.7)	269	
Don’t know	31	(67.9)	14	(32.1)	45	
Education						0.12
None	381	(47.4)	422	(52.6)	802	
Primary	896	(40.9)	1296	(59.1)	2191	
Secondary and above	212	(43.1)	280	(56.9)	492	
History of previous pregnancy						0.12
No	371	(39.3)	568	(60.7)	939	
Yes	1113	(43.8)	1426	(56.2)	2538	
Number of visit at this facility for this pregnancy						< 0.001
First or second	1194	(48.3)	1280	(51.7)	2474	
Third	191	(30.6)	434	(69.4)	625	
Fourth or more	103	(26.7)	283	(73.3)	386	

### Availability of malaria services for women with none and at least 1 IPTp dose

Additional file 2 presents the weighted frequencies and proportions for variables of availability by dose of IPTp received by women, as indicated on their ANC cards. Women who attended ANC at facilities where diagnosis and treatment of malaria was offered were significantly more likely to have received at least one dose of IPTp than at those where it was not offered (57.3% versus 5.9%; p < 0.01). Women who attended ANC at facilities where more malaria providers diagnosed than prescribed were significantly more likely to have received at least one dose of IPTp (71.4% versus 33.3%, p < 0.01). Women who attended facilities that had SP available on the day of interview were more likely to ever have received IPTp (74.6%) than at facilities that did not (26.3%, p < 0.001). Excluding the IPTp dose received on the day of interview, there was no significant difference in women who had received at least one dose of IPTp versus none in those who attended facilities that had SP available on the day of interview (58.6%) than at facilities that did not (51.4%) (p = 0.17).

### Readiness of facilities to provide malaria services for women with none and at least 1 IPTp dose

Additional file 2 also presents the weighted frequencies and proportions for variables of readiness by dose of IPTp received by women, as indicated on their ANC cards. Women who attended ANC at facilities where training manual and job aid for using malaria RDT were observed were significantly more likely to have received at least one dose of IPTp (66.1% versus 52.3%; p < 0.001). ANC clients who attended facilities where direct observation therapy for uptake of IPTp was routinely practiced were significantly more likely to have received at least one dose of IPTp compared to those at which DOT was not routinely practiced (63.8% versus 46.2%; p < 0.001). Proportion of women who had not received any IPTp dose was much higher for facilities that did not practice DOT than facilities that did (53.9% versus 36.2%). Women who attended facilities with providers who had received any in-service training for malaria (63.3%), ANC training for providing IPTp in the past 2 years (66.9%) or trained for any laboratory services (51.6%) were significantly more likely to have received at least one dose of IPTp. ANC clients who attended facilities at which providers discussed the purpose and side-effects of anti-malarial prophylaxis, and the importance of IPTp doses and using ITNs were significantly more likely to have received at least one dose of IPTp.

### Factors associated with the uptake of IPTp by pregnant women

Table 2 presents the results of univariable and multivariable GEE regression analyses of the factors associated with IPTp uptake. Factors associated with reported use of IPTp by women were facility readiness to provide malaria services, number of visits at the facility for current pregnancy and gestation period. Women who attended ANC at facilities with medium or high readiness scores were more likely to receive IPTp medication than at those with low readiness scores OR = 2.8; 95% CI (1.8–4.3) and OR = 2.1; 95% CI (1.4–3.3), respectively. Women who were attending ANC for the second/third or fourth or more time were significantly more likely to have at least one dose of IPTp (OR = 1.9; 95% CI 1.4–2.6 and OR = 1.8; 95% CI 1.2–2.9, respectively). Women in their third trimester were more likely to have had at least one dose of IPTp (OR = 2.6; 95% CI (2.1–3.3)). Women who attended facilities which reported SP stock out on the day of interview had lower odds of receiving IPTp dose (OR 0.09; 95% CI 0.07–0.1) than those who attended ANC at facilities that did not report stock out. Education level was not statistically associated with IPTp uptake. The difference in women’s uptake of IPTp by managing authority was not statistically significant. Other factors such as availability of malaria services and equipment, maternal age and zones were not associated with IPTp uptake.

**Table 2:** GEE logistic regression of factors associated with the uptake of IPTp by pregnant women

	Univariable		Multivariable	
	OR	(95% CI)	OR	(95% CI)
Education level				
None (ref)		1	1	
Primary	1.3	(0.9–1.9)	1.2	(0.8–2.0)
Secondary and above	1.2	(0.7–2.0)	1.0	(0.6–1.8)
Age				
15–24 (ref)		1		
25–35	1.2	(0.9–1.5)	1.1	(0.9–1.4)
35 and above	1.1	(0.7–1.6)	1.1	(0.8–1.6)
History of previous pregnancy				
Yes		1		
No	1.2	(0.9–1.5)		
Number of visits at this facility for this pregnancy				
First (ref)		1		1
Second or third	1.9***	(1.4–2.4)	1.9**	(1.4–2.6)
Fourth or more	2.3***	(1.6–3.2)	1.8**	(1.2–2.9)
Availability score				
Low (ref)		1		1
Medium	1.2	(0.9–1.7)	0.7	(0.4–1.0)
High	1.2	(0.9–1.7)	0.8	(0.5–1.1)
Readiness score				
Low (ref)		1		1
Medium	4.9***	(3.4–7.0)	2.8***	(1.8–4.3)
High	3.3***	(2.2–4.9)	2.1***	(1.4–3.3)
Managing authority				
Public (ref)		1		1
Private	1.1	(0.7–1.7)	1.4	(0.9–2.2)
Zones				
Central (ref)		1		
Coastal zone	1.0	(0.6–1.7)		
Lake zone	1.1	(0.7–1.9)		
Northern zone	1.0	(0.6–1.7)		
Southern highlands	1.0	(0.6–1.8)		
Western zone	0.6	(0.3–1.3)		
Trimester of gestation				
2nd trimester (ref)		1		1
3rd trimester	2.2***	(1.8–2.6)	2.6***	(2.1–3.3)
Facility location				
Urban (ref)		1		1
Rural	0.7	(0.4–1.1)	0.9	(0.5–1.5)
SP stock out				
Available (ref)		1		1
Not available today	0.09***	(0.07–0.1)	0.09***	(0.07–0.1)
Never available	0.06***	(0.01–0.4)	0.08***	(0.01–0.5)
Facility type				
Private (ref)		1		1
National referral/regional	1.1	(0.6–2.0)	0.9	(0.4–1.8)
District hospital	0.8	(0.5–1.3)	1.7	(0.9–3.3)
Health centre	0.6*	(0.4–0.9)	0.8	(0.6–1.3)
Clinic/dispensary	0.5**	(0.3–0.8)	0.9	(0.5–1.4)

***p < 0.001, **p < 0.01, *p < 0.05

## Discussion

The proportion of women in their second and third trimester who had at least two doses of IPTp as indicated on their card were 7.6% and 32.9%, respectively; for at least one dose, they were 49.2% and 68.3%. This shows that despite of improvements in uptake of IPTp among pregnant women in Tanzania, the proportion is still far from the national target of 80% of the women having the WHO-recommended three doses [14]. These findings further suggest that women tend to take IPTp dose more often in the third trimester than second in spite of the recommendation that a dose must be initiated earliest possible in the second trimester [2]. These results are consistent with other studies done in Tanzania [33–35] which suggested generally lower uptake of IPTp, i.e., non-compliance with IPTp guidelines. The increase of IPTp uptake between trimesters could be because of increased number of ANC visits in the last trimester or poor knowledge regarding the importance of early uptake of IPTp by pregnant women [33]. Tanzania’s National Guidelines for Diagnosing and Treatment of Malaria recommend that the first dose of IPTp should be given at 20–24 weeks and second dose at 28–32 weeks of pregnancy [36]. This differs from WHO recommendations encouraging provision of IPTp starting the 13th week (second trimester) of pregnancy [12]. This also suggests plenty of missed opportunities for benefitting from this important health intervention.

Nationally, the readiness to provide malaria services was about the same (68% and 60% at public and private facilities, respectively), but availability of essential malaria services including diagnosis, was much lower in public facilities (40%) compared to 58% in private facilities. A greater proportion of public facilities (60%) scored low for availability than private (40%). Barriers for overall low availability of malaria services need to be studied further. The variation of health system across zones is highlighted in a study [37], which reported Western zone having insufficient health financing, low coverage of care at birth services for rural women; Western and Central zones also had lower health workforce. In the present study, despite Central and Western zones having slightly lower readiness score than the other zones, the overall score disparities were non-significant.

Factors significantly associated with IPTp uptake were readiness of health facility to provide malaria related services, number of ANC visits, trimester of gestation, and SP stock out. Women attending health facilities with high readiness scores had 2.1 (95% CI 1.4–3.3) times higher odds of uptake of IPTp dose than those who attended facilities with low readiness scores. Based on the scores that were developed, basic services that were positively linked with women’s higher uptake of IPTp include taking of IPTp while directly observed, receiving IPTp during consultation, and attending a facility where provider explained the purpose of, how to take, the effect and the importance of anti-malarial drugs. These results show that creating a health facility environment with increased malaria service readiness results in increased uptake of IPTp.

This study further confirms what other studies reported on the positive link between uptake of IPTp dose and providers’ counselling on the importance of malaria drugs, the danger of malaria in pregnancy [35] and client to re-visit the same clinic, leading to more visits and increasing the likelihood that women will take IPTp doses as provided during visits. A study in Kenya [28] reported that women whose source of health information was a health provider were 12.7 times more likely to receive IPTp than those who relied on other sources, primarily because clearer and more authoritative messages are delivered by providers to the women. Apart from facility readiness, quality of the health services delivered at the facility may have contributed to the increased access to ANC and, therefore, IPTp. Findings from same survey (SPA) conducted in Haiti has shown a strong association between utilization of the primary care including ANC and both infrastructural and service quality especially in rural areas [38], quality items such as provider competency and whether providers deliver adequate health information to the clients contributed to the clients decision for healthcare utilization [38]. In this study, the same items were also found to be associated with IPTp uptake, suggesting that women may choose to utilize ANC not only because of the facility readiness but also based on the perceived quality of care.

SP stock out was negatively associated with uptake of IPTp, thus highlighting the need for inventory management, especially at publicly managed facilities. The finding that health facility readiness is associated with uptake of IPTp underlines the need for government and private institutions providing ANC services to strengthen the essential parameters of readiness for malaria services such as ensuring utilization of training manual and job aid for using malaria RDT, routine in-service training to the malaria service providers and to maintain the policies of DOT and IPTp provision during consultation practices.

Women in their third trimester had higher likelihood of having received IPTp dose than second; one possible reason for this could be that women further in their pregnancy visited ANC more frequently than those in their second trimester, thus increasing the likelihood of receiving IPTp. Common reasons that women have highlighted for not starting ANC earlier in their pregnancy were difficulties in accessing health facilities lower the chances of the four recommended visits [9]. This was also evident when doses given on the day of visit were excluded, there was a 20% decrease in the proportion of women who had received at least 1 dose of IPTp; possibly because the more times a woman attends ANC, the higher the likelihood of having at least one IPTp dose. Managing authority, history of previous pregnancy, women’s age, zones and score of availability of malaria services were not significantly associated with the uptake of IPTp dose among women attending ANC.

## Strengths and limitations

This study has major strengths, one being use of information on availability and readiness of malaria services from the country representative sample of health facilities that were collected from TSPA survey, as well as having actual ANC card data and observing clinic visits. Although there was some nonresponse to the TSPA, the availability of data from the responding facilities was extremely high. Despite of few limitations, this study unlike most of the population-based studies which report on demand side, using TSPA data, reports from provider side (health facilities and providers) where most of the data were based on the actual observations and not self-reported information which are prone to recall bias.

Despite the strengths, the findings of this study should be interpreted with caution because of the following; the actual uptake of IPTp may have been underestimated since this study used data from ANC cards of pregnant women currently attending clinic of which some would still be expecting to receive more doses by end of pregnancy. This study could also not adjust for other factors such as marital status, knowledge of malaria and IPTp, distance to the health facility and socio-economic status of the household that were found to be associated with IPTp uptake in previous studies as well as perceived quality of services at the facility which was also linked with health facility utilization.

## Implications

The national policy targets 80% of all women attending ANC to receive two doses of IPTp. We have observed less than half the target receives two doses of IPTp. Although the national policy clearly indicates proper IPTp schedules, NMCP will need to scale up DOT, training of healthcare workers as well as ensuring drug availability. In order to account for linkage between readiness and IPTP uptake, providers can use guiding materials such as supervisory checklists, prioritize IPTp provision during trainings and strategically plan ANC counselling. These findings highlight key messages which should guide IPTp policy review aiming at reaching the national target. Furthermore, future studies should focus on understanding factors which lead to disparities in supply chain for IPTp as well as qualitative studies on understanding factors influencing IPTp uptake in the community.

## Conclusion

In conclusion, IPTp services and uptake are inadequate. There are disparities in malaria service availability and readiness across geographical location and managing authorities. IPTp uptake was determined by the number of visits to ANC, gestational age and facility readiness in providing IPTp.

## Additional files

**Additional file 1.** Weighted Frequencies and Proportions of Malaria services measuring Availability and Readiness by Managing Authority.

**Additional file 2.** Weighted Frequencies and Proportions of Malaria services measuring Availability and Readiness by uptake of IPTp dose in the second and third trimester.

## Data Availability

SPA data is available upon request from the DHS website.

## Abbreviations

ANC: antenatal care
DHS: Demographic Health Survey
DOT: directly observed therapy
GEE: generalized estimating equation
HIV: human immunodeficiency virus
IPTp: intermittent preventive treatment during pregnancy
ITN: insecticide treated nets
MIS: Malaria Indicator Survey
NMCP: National Malaria Control Programme
PCA: principal component analysis
PNC: post-natal care
RDT: rapid diagnostic test
SARA: Service Availability and Readiness Assessment
SP: sulphadoxine pyrimethamine
SPA: Service Provision Assessment
TSPA: Tanzania Service Provision Assessment
WHO: World Health Organization
