# Supplementary material for: Assessment of the impact of availability and readiness of malaria services on uptake of intermittent preventive treatment in pregnancy (IPTp) provided during ANC visits in Tanzania
Source: Malar J. 2019 Jul 9;18:229. doi: 10.1186/s12936-019-2862-3 (PMC6617666; doi:10.1186/s12936-019-2862-3)
Supplement: Supplementary file 1 — Additional file 1. Weighted Frequencies and Proportions of Malaria services measuring Availability and Readiness by Managing Authority. [file 12936_2019_2862_MOESM1_ESM.docx]

**Additional files**

**Additional file 1 Weighted Frequencies and Proportions of Malaria services measuring Availability and Readiness by Managing Authority**

|  | Public | | Private | | Overall | | p-value |
| --- | --- | --- | --- | --- | --- | --- | --- |
|  | n | (%) | n | (%) | n | (%) |  |
| AVAILABILITY |  |  |  |  |  |  |  |
| Number of facilities | **797** | **(81.8%)** | **176** | **(14.2%)** | **973** |  |  |
| Facility Offers any Malaria tests | 692 | (86.8) | 138 | (78.1) | 830 | (85.2) | 0.06 |
| Facility offers Malaria RDT | 688 | (86.2) | 125 | (71.0) | 813 | (83.5) | 0.001 |
| Facility offers Malaria Lab Test | 77 | (9.6) | 58 | (33.0) | 135 | (13.8) | <0.001 |
| Facility offers Malaria Diagnosis/Treatment | 793 | (99.5) | 176 | (99.7) | 969 | (99.5) | 0.57 |
| SP stock at the facility |  |  |  |  |  |  | 0.15 |
| Never available | 7 | (0.9) | 6 | (3.3) | 13 | (1.4) |  |
| Available | 466 | (58.4) | 98 | (55.8) | 564 | (57.9) |  |
| Not available today | 324 | (40.7) | 72 | (40.9) | 396 | (40.7) |  |
| Malaria providers diagnose and/or prescribe treatment |  |  |  |  |  |  | 0.87 |
| Diagnose and Prescribe | 766 | (96.1) | 168 | (95.4) | 934 | (96.0) |  |
| Diagnose only | 31 | (3.9) | 8 | (4.6) | 39 | (4.0) |  |
| Total number of health care providers | **4177** | **(63.1%)** | **2441** | **(36.9%)** | **6618** |  |  |
| Provider provides diagnosis or treatment of Malaria | 3243 | (77.6) | 1487 | (60.9) | 4730 | (71.4) | <0.001 |
| Provider provides ANC/PNC care | 3166 | (75.8) | 988 | (40.5) | 4154 | (62.8) | <0.001 |
| Provider provides any lab services | 986 | (23.6) | 591 | (24.2) | 1577 | (23.8) | 0.70 |
| Provider provides Malaria Microscopy | 332 | (33.7) | 452 | (76.5) | 784 | (49.7) | <0.001 |
| Provider provides Malaria RDT | 871 | (88.4) | 498 | (84.4) | 1370 | (86.9) | 0.24 |
| Qualifications of health care providers |  |  |  |  |  |  | <0.001 |
| Medical doctors | 161 | (3.9) | 187 | (7.7) | 347 | (5.3) |  |
| Clinicians | 836 | (20.1) | 424 | (17.4) | 1260 | (19.0) |  |
| Nurse professionals | 2861 | (68.5) | 1393 | (57.1) | 4254 | (64.3) |  |
| Lab personnel | 319 | (7.6) | 432 | (17.7) | 751 | (11.3) |  |
| ANC observations and client exit interview | **2815** | **(80.5%)** | **681** | **(19.5%)** | **3496** |  |  |
| Number of visits at this facility for this pregnancy |  |  |  |  |  |  | <0.001 |
| First or second | 2018 | (71.7) | 467 | (68.6) | 2485 | (46.4) |  |
| Third | 525 | (18.6) | 100 | (14.7) | 625 | (17.9) |  |
| Fourth or more | 272 | (9.7) | 114 | (16.7) | 386 | (11.0) |  |
| Qualifications for ANC |  |  |  |  |  |  | <0.001 |
| Medical doctors | 10 | (0.3) | 15 | (2.4) | 25 | (0.7) |  |
| Clinicians | 183 | (6.5) | 9 | (1.3) | 192 | (5.5) |  |
| Nurse professionals | 2623 | (93.2) | 656 | (96.4) | 3279 | (93.8) |  |
| Facility nearest to home | 2524 | (89.7) | 536 | (78.7) | 3060 | (87.5) | <0.001 |
| READINESS |  |  |  |  |  |  |  |
| Number of facilities | **n=797** | **(81.8%)** | **176** | **(14.2%)** | **973** |  |  |
| Training manual/ Job aid for using Malaria RDT | 210 | (26.3) | 76 | (43.2) | 286 | (29.3) | 0.001 |
| Uptake of IPTp directly observed at Facility | 530 | (66.4) | 123 | (70.0) | 653 | (67.0) | 0.56 |
| National guideline for Treatment of Malaria observed | 501 | (62.8) | 87 | (49.4) | 588 | (60.4) | 0.03 |
| Number of all providers | **4177** | **(63.1%)** | **2441** | **(36.9%)** | **6618** |  |  |
| Any in-service training for Malaria | 1829 | (43.8) | 686 | (28.1) | 2515 | (38.0) | <0.001 |
| In-service training for diagnosing Malaria in adults | 1327 | (72.5) | 549 | (80.0) | 1876 | (74.6) | 0.03 |
| In-service training for providing IPTp to pregnant women | 1299 | (71.0) | 481 | (70.0) | 1781 | (70.8) | 0.95 |
| Training on how to perform Malaria Microscopy | 148 | (8.1) | 130 | (18.9) | 278 | (11.1) | <0.001 |
| Total number of providers ever trained for ANC | **3853** |  | **2011** |  | **5864** |  |  |
| Among those, number who were trained on: |  |  |  |  |  |  |  |
| In-service training for ANC | 1188 | (30.8) | 394 | (19.6) | 1582 | (26.9) | <0.001 |
| Providing IPTp | 935 | (78.7) | 297 | (75.3) | 1232 | (77.8) | 0.04 |
| Any lab services | 591 | (59.9) | 335 | (56.6) | 926 | (58.7) | 0.46 |
| Malaria Microscopy | 102 | (17.3) | 124 | (37.0) | 227 | (24.5) | <0.001 |
| Malaria RDT | 408 | (69.1) | 230 | (68.7) | 638 | (68.9) | 0.29 |
| ANC observations and client exit interview | **2815** | **(80.5%)** | **681** | **(19.5%)** | **3496** |  |  |
| Routinely provide Malaria RDT at ANC |  |  |  |  |  |  | 0.77 |
| Observed | 763 | (27.1) | 181 | (26.6) | 944 | (27.0) |  |
| Reported, not observed | 133 | (4.7) | 45 | (6.7) | 179 | (5.1) |  |
| Provider discussed importance of at least 4 ANC visits | 471 | (16.7) | 111 | (16.3) | 582 | (16.7) | 0.90 |
| Provider gave SP during consultation | 987 | (35.1) | 216 | (31.8) | 1203 | (34.4) | 0.49 |
| Provider explained purpose of preventive anti-malarial | 1137 | (40.4) | 229 | (33.6) | 1366 | (39.1) | 0.13 |
| Provider explained how to take anti-malarial | 866 | (30.8) | 148 | (21.7) | 1014 | (28.9) | 0.03 |
| Provider explained side effects of anti-malarial | 103 | (3.7) | 13 | (1.8) | 116 | (3.3) | 0.04 |
| Interviewer observed first IPTp uptake in presence of provider | 527 | (18.7) | 149 | (21.9) | 676 | (19.3) | 0.42 |
| Provider explained importance of further IPTp doses | 196 | (7.0) | 42 | (6.2) | 239 | (6.8) | 0.69 |
| Provider explained importance of using ITN | 215 | (7.7) | 38 | (5.6) | 253 | (7.2) | 0.31 |
