# Supplementary material for: Assessment of the impact of availability and readiness of malaria services on uptake of intermittent preventive treatment in pregnancy (IPTp) provided during ANC visits in Tanzania
Source: Malar J. 2019 Jul 9;18:229. doi: 10.1186/s12936-019-2862-3 (PMC6617666; doi:10.1186/s12936-019-2862-3)
Supplement: Supplementary file 2 — Additional file 2. Weighted Frequencies and Proportions of Malaria services measuring Availability and Readiness by uptake of IPTp dose in the second and third trimester. [file 12936_2019_2862_MOESM2_ESM.docx]

**Additional file 2 Weighted Frequencies and Proportions of Malaria services measuring Availability and Readiness by uptake of IPTp dose in the second and third trimester.**

|  | None | | At least 1 dose of IPTp | | Overall | p-value |
| --- | --- | --- | --- | --- | --- | --- |
| AVAILABILITY | **n** | **(%)** | **n** | **(%)** |  |  |
| Facility |  |  |  |  |  |  |
| Facility Offers any Malaria tests |  |  |  |  |  | 0.57 |
| Yes | 1251 | (42.0) | 1726 | (57.9) | 2977 |  |
| No | 237 | (46.7) | 271 | (53.4) | 508 |  |
| Facility offers Malaria RDT |  |  |  |  |  | 0.77 |
| Yes | 1229 | (42.3) | 1674 | (57.7) | 2903 |  |
| No | 259 | (44.5) | 322 | (55.5) | 581 |  |
| Facility offers Malaria Lab Test |  |  |  |  |  | 0.11 |
| Yes | 359 | (37.8) | 591 | (62.2) | 950 |  |
| No | 1129 | (44.5) | 1406 | (55.5) | 2534 |  |
| Facility offers Malaria Diagnosis/Treatment |  |  |  |  |  | <0.01 |
| Yes | 1485 | (42.7) | 1997 | (57.3) | 3482 |  |
| No | 2 | (94.1) | 0.14 | (5.9) | 2 |  |
| Malaria providers diagnose and/or prescribe treatment |  |  |  |  |  | <0.01 |
| Diagnose and Prescribe | 1455 | (43.1) | 1921 | (56.9) | 3375 |  |
| Diagnose only | 30 | (28.6) | 74 | (71.4) | 104 |  |
| Prescribe only | 4 | (66.7) | 2 | (33.3) | 5 |  |
| SP stock |  |  |  |  |  | <0.001 |
| Never available | 31 | (2.1) | 15 | (0.8) | 46 |  |
| Available | 391 | (26.3) | 1490 | (74.6) | 1881 |  |
| Not available today | 1066 | (71.6) | 492 | (24.6) | 1557 |  |
| Malaria Providers |  |  |  |  |  |  |
| Provider provides diagnosis or treatment of Malaria |  |  |  |  |  | 0.23 |
| Yes | 1233 | (43.7) | 1589 | (56.3) | 662 |  |
| No | 254 | (38.4) | 408 | (61.6) | 2823 |  |
| Provider provides any lab services |  |  |  |  |  | 0.77 |
| Yes | 169 | (41.1) | 241 | (58.9) | 410 |  |
| No | 1319 | (42.9) | 1756 | (57.1) | 3075 |  |
| Provider provides Malaria Microscopy |  |  |  |  |  | 0.14 |
| Yes | 2 | (33.3) | 4 | (66.7) | 6 |  |
| No | 167 | (41.3) | 237 | (58.8) | 404 |  |
| Provider provides Malaria RDT |  |  |  |  |  | 0.87 |
| Yes | 127 | (40.6) | 186 | (59.4) | 312 |  |
| No | 42 | (42.9) | 56 | (57.1) | 98 |  |
| ANC observations and client exit interview |  |  |  |  |  |  |
| Facility nearest to home |  |  |  |  |  | 0.81 |
| Yes | 1304 | (42.8) | 1745 | (57.2) | 3052 |  |
| No | 183 | (42.2) | 251 | (57.8) | 434 |  |
| READINESS |  |  |  |  |  |  |
| Facility |  |  |  |  |  |  |
| Training manual/ Job aid for using Malaria RDT observed |  |  |  |  |  | <0.001 |
| Yes | 437 | (33.9) | 850 | (66.1) | 1286 |  |
| No | 1038 | (47.7) | 1138 | (52.3) | 2176 |  |
| National guideline for Diagnosis and Treatment of Malaria observed |  |  |  |  |  | 0.40 |
| Yes | 878 | (41.2) | 1252 | (58.8) | 2131 |  |
| No | 610 | (45.0) | 744 | (54.9) | 1354 |  |
| Uptake of IPTp directly observed at Facility |  |  |  |  |  | <0.001 |
| Yes | 795 | (36.2) | 1403 | (63.8) | 2198 |  |
| No | 693 | (53.9) | 594 | (46.2) | 1287 |  |
| Malaria Providers |  |  |  |  |  |  |
| Any in-service training for Malaria |  |  |  |  |  | <0.01 |
| Yes | 576 | (36.7) | 994 | (63.3) | 1570 |  |
| No | 912 | (47.7) | 1003 | (52.3) | 1915 |  |
| In-service training for diagnosing Malaria in adults the past 2 years |  |  |  |  |  | 0.47 |
| Yes | 128 | (32.6) | 266 | (67.4) | 394 |  |
| No | 186 | (35.2) | 342 | (64.8) | 528 |  |
| In-service training for treating Malaria in pregnancy in the past 2 years |  |  |  |  |  | 0.54 |
| Yes | 143 | (31.7) | 309 | (68.4) | 452 |  |
| No | 161 | (38.4) | 259 | (61.6) | 420 |  |
| Training on how to perform Malaria Microscopy in the past 2 years |  |  |  |  |  | 0.50 |
| Yes | 8 | (21.6) | 28 | (78.4) | 36 |  |
| No | 559 | (37.3) | 942 | (62.8) | 1501 |  |
| Training on how to perform Malaria RDT in the past 2 years |  |  |  |  |  | 0.64 |
| Yes | 134 | (34.6) | 254 | (65.4) | 388 |  |
| No | 147 | (34.0) | 284 | (65.9) | 431 |  |
| Any in-service training for ANC |  |  |  |  |  | 0.94 |
| Yes | 641 | (42.5) | 866 | (57.4) | 1507 |  |
| No | 847 | (42.8) | 1131 | (57.2) | 1978 |  |
| ANC training for providing IPTp in the past 2 years |  |  |  |  |  | 0.05 |
| Yes | 111 | (33.0) | 224 | (66.9) | 335 |  |
| No | 167 | (52.1) | 153 | (47.9) | 320 |  |
| Trained for any lab services |  |  |  |  |  | 0.02 |
| Yes | 132 | (48.4) | 141 | (51.6) | 273 |  |
| No | 36 | (26.6) | 101 | (73.4) | 137 |  |
| Trained on Malaria RDT in the past 2 years |  |  |  |  |  | 0.35 |
| Yes | 59 | (60.2) | 39 | (39.8) | 99 |  |
| No | 50 | (40.5) | 74 | (59.5) | 124 |  |
| ANC observations and client exit interview |  |  |  |  |  |  |
| Provider advised use of ITNs |  |  |  |  |  | <0.001 |
| Yes, this and previous visit | 99 | (25.5) | 290 | (74.5) | 389 |  |
| Yes, only this visit | 291 | (45.0) | 355 | (55.0) | 646 |  |
| Yes, only previous visit | 123 | (31.0) | 273 | (68.9) | 396 |  |
| Provider discussed importance of at least 4 ANC visits |  |  |  |  |  | 0.69 |
| Yes | 241 | (41.3) | 342 | (58.7) | 582 |  |
| No | 1247 | (43.0) | 1655 | (57.0) | 2902 |  |
| Provider gave SP during consultation |  |  |  |  |  | <0.001 |
| Yes | 54 | (4.5) | 1149 | (95.5) | 1203 |  |
| No | 1433 | (62.8) | 848 | (37.1) | 2281 |  |
| Provider explained purpose of preventive anti-malarial |  |  |  |  |  | <0.001 |
| Yes | 292 | (21.5) | 1067 | (78.5) | 1359 |  |
| No | 1196 | (56.3) | 930 | (43.7) | 2126 |  |
| Provider explained how to take anti-malarial |  |  |  |  |  | <0.001 |
| Yes | 123 | (12.2) | 884 | (87.8) | 1006 |  |
| No | 1365 | (55.1) | 1113 | (44.9) | 2479 |  |
| Provider explained side effects of anti-malarial |  |  |  |  |  | <0.001 |
| Yes | 12 | (10.6) | 104 | (89.4) | 116 |  |
| No | 1475 | (43.9) | 1893 | (56.2) | 3369 |  |
| Interviewer observed first IPTp uptake in presence of provider |  |  |  |  |  | <0.001 |
| Yes | 31 | (4.6) | 645 | (95.5) | 676 |  |
| No | 1457 | (51.9) | 1352 | (48.1) | 2809 |  |
| Provider explained importance of further IPTp doses |  |  |  |  |  | <0.001 |
| Yes | 17 | (6.9) | 222 | (93.1) | 239 |  |
| No | 1471 | (45.3) | 1775 | (54.7) | 3246 |  |
| Provider explained importance of using ITN |  |  |  |  |  | 0.08 |
| Yes | 84 | (33.0) | 170 | (66.9) | 253 |  |
| No | 1404 | (43.5) | 1827 | (56.6) | 3231 |  |
